# Supplementary material for: Modelling the effects of environmental heterogeneity within the lung on the tuberculosis life-cycle
Source: J Theor Biol. 2020 Dec 7;506:110381. doi: 10.1016/j.jtbi.2020.110381 (PMC7511696; doi:10.1016/j.jtbi.2020.110381)
Supplement: Supplementary file 1 [file mmc1.pdf]

Modelling the effects of environmental heterogeneity within the lung on the tuberculosis life-cycle - Supplementary Material

Michael J. Pitcher, Ruth Bowness, Simon Dobson,
Raluca Eftimie, Stephen H. Gillespie

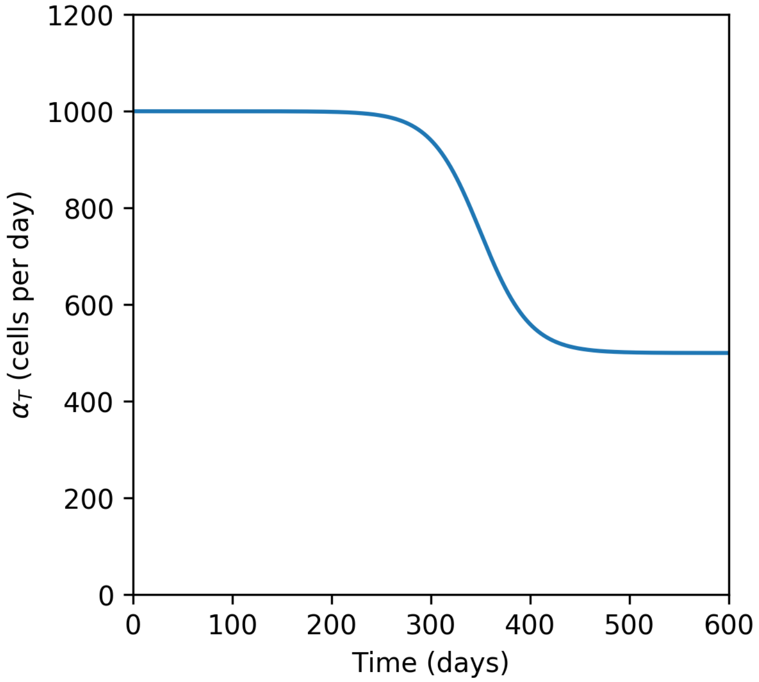

Figure S1: Change in T cell recruitment rate (α_T) over time, when $\alpha_T(0) = 1000$

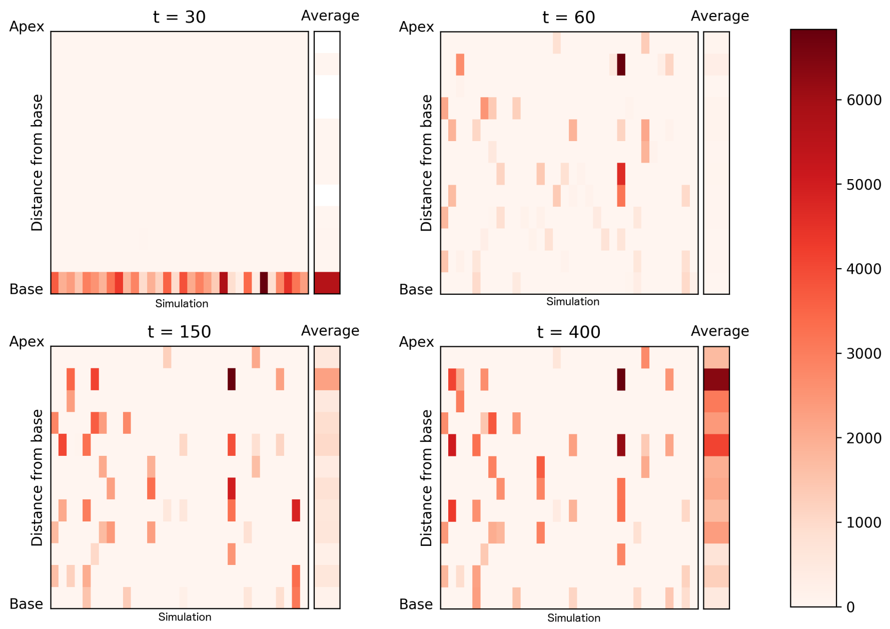

Figure S2: Average lesion size (number of bacteria) at each of 12 evenly sized horizontal slices of lung at different time-steps during the simulation. Columns represent individual simulations, with the final column representing the average across simulations.

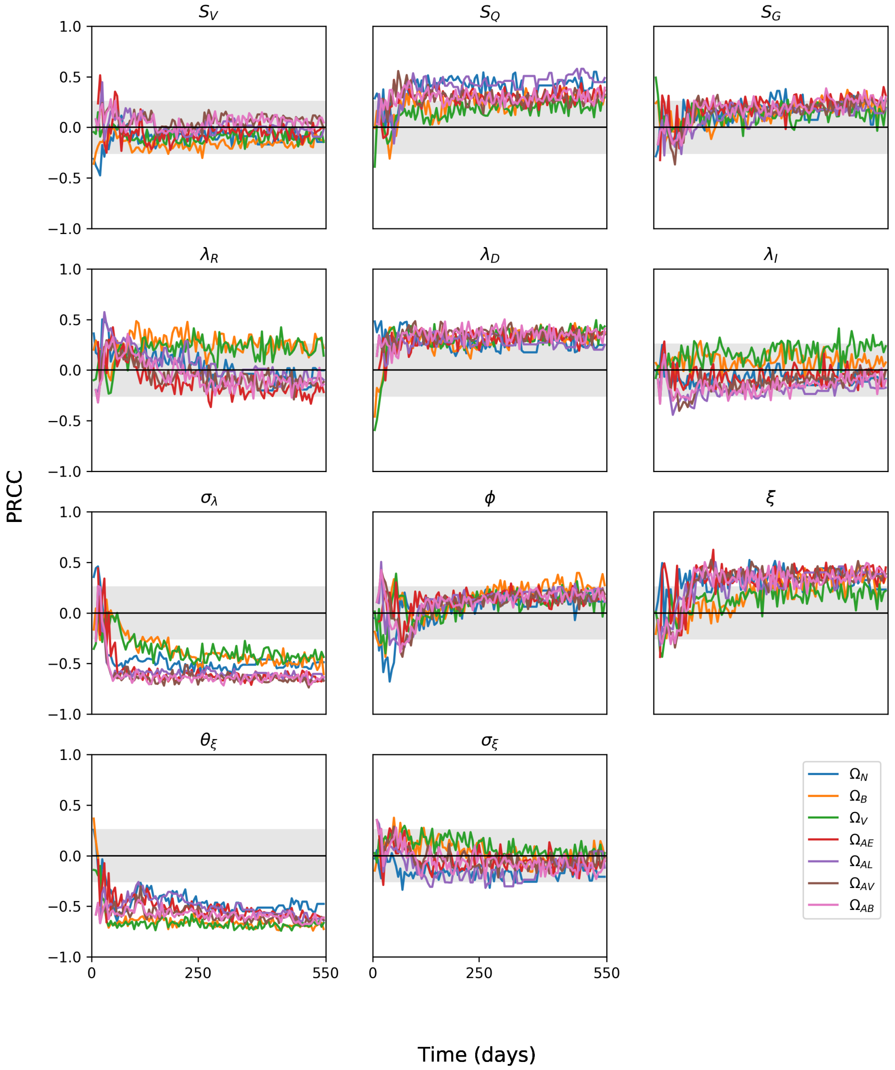

Figure S3: Sensitivity plots for various parameters within *TBMetapopPy*, plotting the PRCC value of the parameter against the model outputs over time. Grey shaded area shows non-significance (p<0.01).

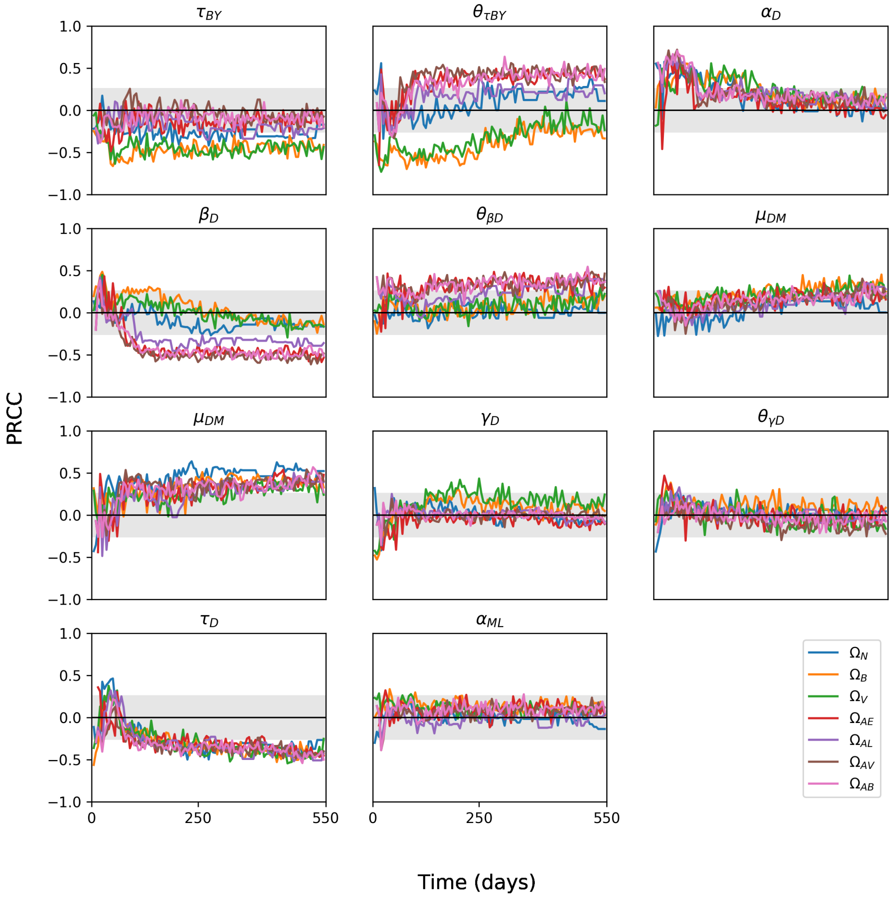

Figure S4: Sensitivity plots for various parameters within *TBMetapopPy*, plotting the PRCC value of the parameter against the model outputs over time. Grey shaded area shows non-significance (p<0.01).

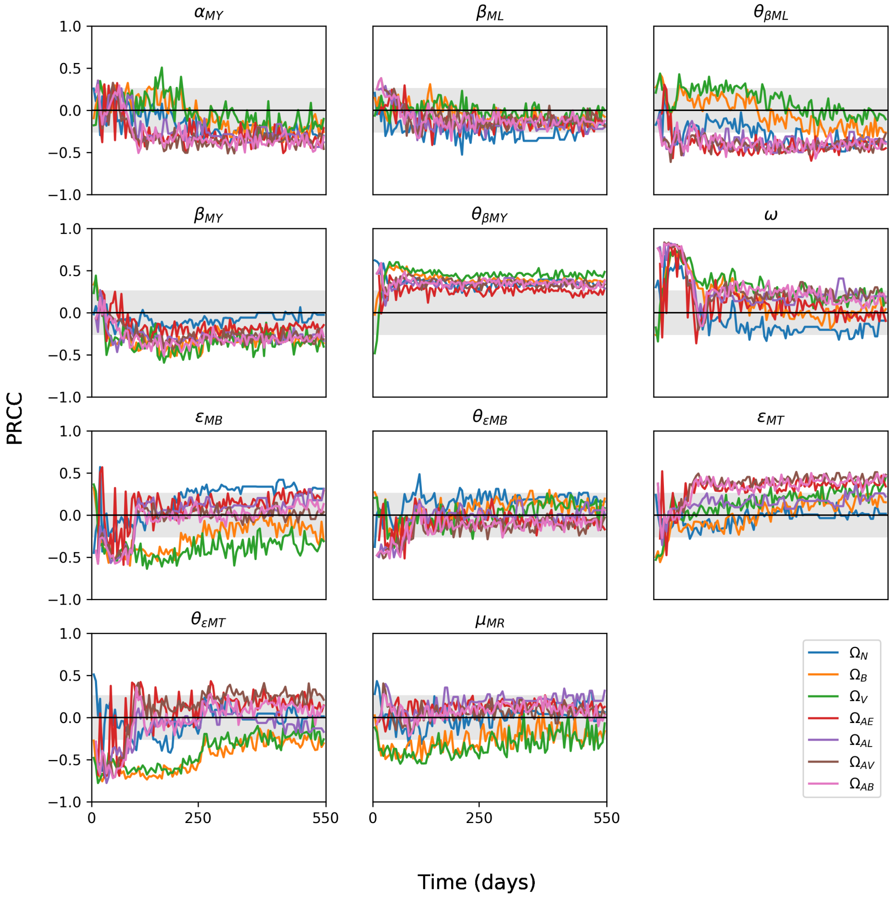

Figure S5: Sensitivity plots for various parameters within *TBMetapopPy*, plotting the PRCC value of the parameter against the model outputs over time. Grey shaded area shows non-significance ($p<0.01$).

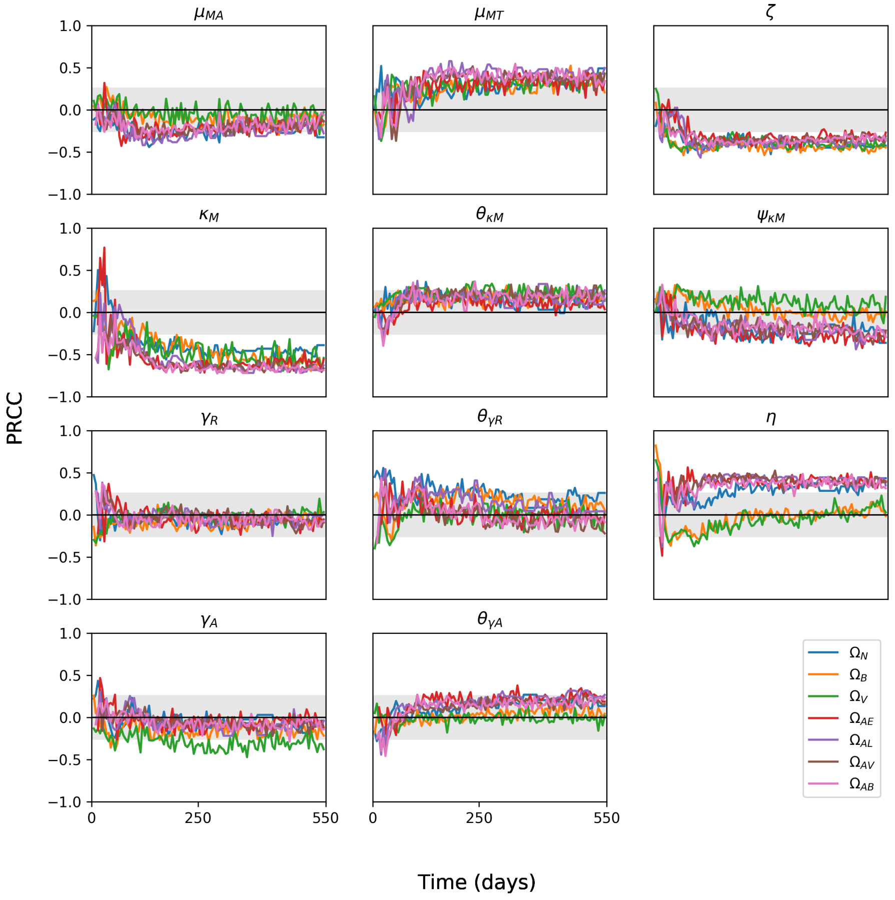

Figure S6: Sensitivity plots for various parameters within *TBMetapopPy*, plotting the PRCC value of the parameter against the model outputs over time. Grey shaded area shows non-significance ($p<0.01$).

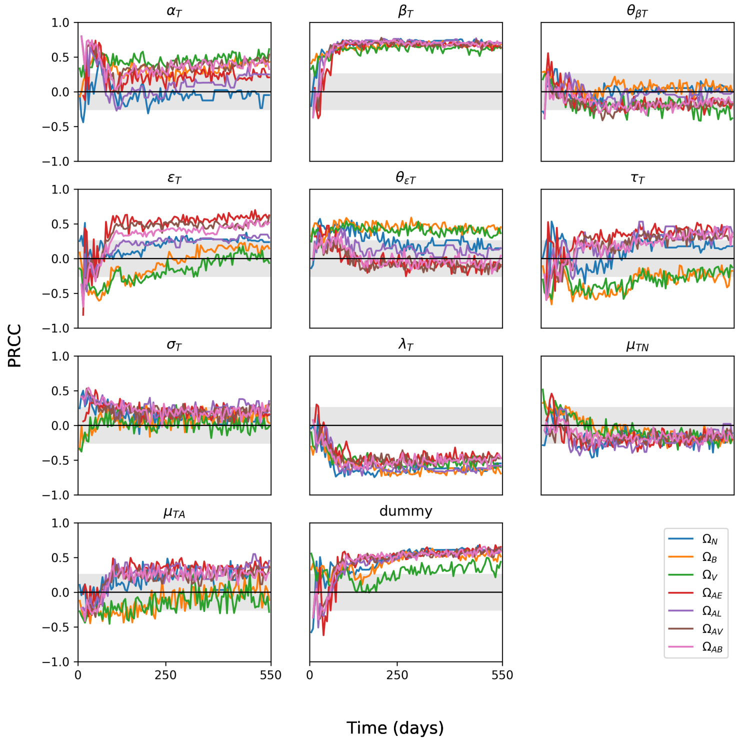

Figure S7: Sensitivity plots for various parameters within *TBMetapopPy*, plotting the PRCC value of the parameter against the model outputs over time. Grey shaded area shows non-significance ($p<0.01$).
